# Renal Function Outcomes and Risk Factors for Risk Factors for Stage 3B Chronic Kidney Disease after Urinary Diversion in Patients with Muscle Invasive Bladder Cancer

**DOI:** 10.1371/journal.pone.0149544

**Published:** 2016-02-22

**Authors:** Shingo Hatakeyama, Takuya Koie, Takuma Narita, Shogo Hosogoe, Hayato Yamamoto, Yuki Tobisawa, Tohru Yoneyama, Takahiro Yoneyama, Yasuhiro Hashimoto, Chikara Ohyama

**Affiliations:** 1 Department of Urology, Hirosaki University Graduate School of Medicine, Hirosaki, Japan; 2 Department of Advanced Transplant and Regenerative Medicine, Hirosaki University Graduate School of Medicine, Hirosaki, Japan; University of Louisville, UNITED STATES

## Abstract

**Objectives:**

To assess the effects of urinary diversion on renal function, we retrospectively investigated renal function over 5 years after urinary diversion using a propensity score matching strategy.

**Methods:**

Between May 1996 and November 2013, 345 consecutive adult patients underwent radical cystectomy and urinary diversion in our hospital; one hundred and fifteen patients with more than a 5-year follow-up were enrolled. Propensity scores were calculated using logistic analysis, and the data used in the analyses included age, gender, Eastern Cooperative Oncology Group Performance Status (ECOG-PS), clinical tumor stage, presence of cardiovascular disease; hypertension; and type 2 diabetes and preoperative eGFR at the initial visit. Multivariate logistic regression analysis was used to assess the risk factors for stage 3B chronic kidney disease (CKD) after the different types of urinary diversion.

**Results:**

Continent and incontinent diversion were performed in 68 and 47 patients, respectively. The mean preoperative eGFR was significantly lower in the incontinent than in the continent group (*P* < 0.001). In propensity score-matched patients (*n* = 34 each), no significant differences were observed in pre- and postoperative eGFR and 5-year eGFR decrease rates between the groups. In the incontinent group, the number of postoperative stage 3B CKD patients was significantly increased than the continent group. Using multivariate analysis, independent risk factors significantly associated with stage 3B CKD at 5 years after surgery were older age, eGFR before surgery, incontinent diversion (cutaneous ureterostomy), and postoperative hydronephrosis.

**Conclusions:**

The types of urinary diversion had no significant impact on renal function decline, whereas older age, preexisting impaired renal function, postoperative hydronephrosis, and cutaneous ureterostomy were independent risk factors for stage 3B CKD at 5 years after radical cystectomy.

## Introduction

Radical cystectomy and urinary diversion remain the standard treatment modality for muscle-invasive bladder cancer patients. However, these are associated with the significant risks of perioperative and long-term morbidity and mortality,[1, 2] including subsequent renal function decline.[3, 4] The goals of urinary diversion after radical cystectomy have evolved from the protection of the upper tracts to functional and anatomical restoration because of the high proportion of bladder cancer patients presenting with impaired renal function.[5] Patients with urinary diversion are notably at a high risk of renal function decline,[6] and with chronic kidney disease (CKD) have a high risk for cardiovascular disease and all-cause mortality.[7] However, limited evidence describing the effects of urinary diversion on renal function after radical cystectomy is available, and results are controversial.[8–14] Recent studies supported choice of urinary diversion was not independently associated with renal function decline. [10–12, 14] Because majority of the clinical reports describing renal function after urinary diversion are reported from Western countries, their conclusions are impeded by differences in patients’ backgrounds. Therefore, their findings need to be confirmed in Japanese people.

In this study, we used propensity score matching methods to balance the treatment groups at baseline and guarantee the validity of this retrospective analysis. Matching subjects using their propensity scores is one method of controlling for measurable confounding factors when numerous characteristics are related to the outcome of interest or when two populations are known to differ due to selection bias.[15]

Using this propensity matching strategy, we compared renal function over 5 years [estimated glomerular filtration rate (eGFR)] after urinary diversion between patients with incontinent and continent urinary diversion and evaluated the predictors associated with worsening renal function in pair-matched patients. The primary endpoint in this study was renal function decline after urinary diversion. Secondary endpoints was risk factors for stage 3B CKD after urinary diversion.

## Materials and Methods

### Patient selection

Between May 1996 and November 2013, 345 consecutive adult patients underwent radical cystectomy and urinary diversion in our department and received ileal conduit diversion, cutaneous ureterostomy, or orthotopic ileal neobladder diversion. Of these, 115 patients with more than 5 years of follow-up were identified. Patients with nephrectomy or who died within 5 years were excluded. In this cohort, we compared pre- and postoperative renal function between patients with continent urinary diversion (orthotopic ileal neobladder: the continent group) and incontinent urinary diversion (ileal conduit or cutaneous ureterostomy: the incontinent group). The decision of urinary diversion and the nerve sparing approach was determined by the surgeon and/or based on the patient’s preference. Impaired renal function (serum creatinine level > 2.0 mg/mL or eGFR < 30 mL/min/1.73 m^2^) was a contraindication for orthotopic ileal neobladder. If the tumor was located in bladder neck in female patient, or invading to prostate in male patient, orthotopic ileal neobladder was not performed. Because we administered neoadjuvant chemotherapy to patients with advanced disease, adjuvant chemotherapy was not given in this cohort.

### Ethics Statement

This study was performed in accordance with the ethical standards of the Declaration of Helsinki, and approved by an ethics review board of Hirosaki University School of Medicine (the authorization number; 2015–047). The participants in this study provide their verbal informed consent, and it was recorded in medical chart. Pursuant to the provisions of the ethics committee and the ethic guideline in Japan, written consent was not required in exchange for public disclosure of study information in the case of retrospective and/or observational study using a material such as the existing documentation. The ethics committees in Hirosaki University School of Medicine approved this consent procedure. The study information was open for the public consumption at http://www.med.hirosaki-u.ac.jp/~uro/html/IRB/IRBdoc.html.

### Pair-matching methods

Because of the retrospective nature of this study, patients’ backgrounds, particularly preoperative eGFR, were significantly different between the groups. Therefore, to guarantee the validity of this retrospective analysis, we used the propensity score matching strategy to compare renal function decline between patients with continent and incontinent diversion. Propensity scores were calculated using logistic analysis, and the data used in the analyses included age, gender, Eastern Cooperative Oncology Group Performance Status (ECOG-PS), clinical tumor stage, presence of cardiovascular disease; hypertension; and type 2 diabetes, and preoperative eGFR at the initial visit. Based on the scores of each patient, two patients with a score within 0.03 were selected as a pair between the continent and incontinent diversion groups.

### Surgical procedure

All patients underwent radical cystectomy, urinary diversion, and lymphadenectomy procedures, which included the removal of the obturator, external iliac, hypogastric, and common iliac lymph node chains (no para-aortic or paracaval dissection). All radical cystectomy presented here were performed by two surgeons (C.O. or T. K.), and the basic technique was identical regardless of the surgeon.[16] Metastatic disease including uncontrollable lymph node involvement, or ECOG-PS > 2 was regarded as a contraindication for radical cystectomy.

In cutaneous ureterostomy, the stoma was created according to the method of Toyoda.[17] In brief, the distal end of the ureter that was passed through the abdominal wall was cut longitudinally to create a fish mouth aperture. The epidermis and dermis were resected from the skin areas corresponding to the separated ureteral segments. The ureteral segments were sutured to the skin to fill the defect using absorbable sutures. A ureteral stent remained indwelling for 1 week after surgery. A ureteral stent was indwelled when repeated febrile pyelonephritis or ureteral stenosis was observed.

In ileal conduit diversion, an ileal segment of 15–20 cm in length was isolated approximately 20 cm proximal to the ileocecal valve. Ureters were split and anastomosed separately using the Nesbit technique[18] in an open end-to-side manner. The ileal segment was anastomosed to the abdominal wall in a nipple-to-stoma manner.

In orthotopic ileal neobladder, ileal reservoir construction was performed according to previously reported methods.[19, 20] In brief, a small bowel segment of 40 cm in length was resected approximately 20 cm proximal to the ileocecal valve. The ileal segment was then split open along the antimesenteric border so that it was detubularized. Small bowel loops were then arranged in a U shape, and the inner opposite borders were oversewn with a single-layer of seromuscular running suture using 3–0 braided polyglactin. Ureteroileal anastomosis was performed bilaterally without an antireflux procedure. A new meatus urethra (8–10 mm in diameter) was created at the bottom of the pouch wall. Four 3–0 braided polyglactin sutures for seromuscular anastomosis were placed between the hole in the reservoir wall and the membranous urethra. An 18-F urethral catheter with 30 cc balloon was inserted before tying the four sutures.

### Patient follow-up

Each patient was assessed every 3 months using ultrasonography to monitor for hydronephrosis, serum electrolytes, blood urea nitrogen, serum creatinine, and liver function. Computed tomography (CT) was performed every 6 months for the early detection of tumor recurrence. Urethroscopic examination was performed at 3-month intervals during 2 years.

#### Evaluation of renal function

Renal function was evaluated using eGFR. The following equation used to estimate eGFR for Japanese patients is a modified version of the abbreviated Modification of Diet in Renal Disease Study formula: eGFR mL/min/1.73 m^2^ = 194 × sCr^−1.094^ × age^−0.287^ (× 0.739, if female).[21] Chronic kidney disease (CKD) stages[22] were also used to classify renal function. Because the mean value of eGFR at 5 years was 59 mL/min/1.73 m^2^, and 47% of patients exhibited eGFR < 60 mL/min/1.73 m^2^ (stage 3 CKD) in this cohort, we defined impaired renal function after surgery as eGFR < 45 mL/min/1.73 m^2^ (stage 3B CKD). Tumor stage and grade were assigned according to the 2009 TNM classification of the Union of International Cancer Control.[23]

#### Evaluation of variables

The variables analyzed were age, gender, ECOG-PS, history of cardiovascular disease; hypertension; and type 2 diabetes, clinical and pathological stage, types of urinary diversion, blood loss, operative duration, postoperative complications, indwelling ureteral stent, tumor recurrence (excluding the upper urinary tract), and eGFR. For eGFR, each patient was evaluated using pre- and postoperative eGFR at 1 month, and 1, 2, 3, and 5 years. Pyelonephritis was defined as a positive urine culture and the presence of flank pain or tenderness with fever (>38.5°C axillary). Surgical site infection, anastomotic leakage of urinary tract, postoperative episodes of acute pyelonephritis, postoperative hydronephrosis, ureteral stricture, urolithiasis, and gastrointestinal complications (ileus, anastomotic leakage) were included in postoperative complications. Presence of postoperative hydronephrosis was evaluated by CT imaging at 12 months after surgery. The hydronephrosis grade was evaluated by CT imaging, and scored according to the hydronephrosis grading scale: grade 0, no dilatation (G0); grade 1, pelvic dilatation only (G1); grade 2, mild caliceal dilatation (G2); grade 3, severe caliceal dilatation (G3); grade 4, renal parenchymal atrophy (G4), as described previously [24, 25]. The hydronephrosis grade 2 or higher was defined as a significant change. Hypertension was defined as taking any antihypertensive medications or preoperative systolic and diastolic blood pressure measurements of >140 and >90 mmHg, respectively. Diabetic patients were defined as those with a history of type 2 diabetes or those who met the relevant diagnostic criteria and required glycemic control.

### Statistical analysis

Statistical analyses of the clinical data were performed using SPSS ver. 19.0 (SPSS, Inc., Chicago, IL, USA) and GraphPad Prism 5.03 (GraphPad Software, San Diego, CA, USA). Categorical variables were compared using the Fisher’s exact test. Quantitative variables were expressed as mean with standard deviation (SD) or median with interquartile range (IQR). The difference between the groups was statistically compared using the Student’s *t*-test for normal distribution or the Mann–Whitney *U*-test for non-normal distribution. *P* values < 0.05 were considered to be statistically significant. Risk factors for eGFR < 45 mL/min/1.73 m^2^ were identified using multivariate analyses with the Cox regression model, and hazard ratios (HRs) with 95% confidence intervals were calculated after controlling simultaneously for potential confounders. Due to the limitation of sample numbers, we included 8 variables in the Cox regression model 1 which were age, comorbidities (history of cardiovascular disease: CVD, hypertension: HTN, or type 2 diabetes: DM), preoperative eGFR, types of urinary diversion (continent or incontinent), postoperative hydronephrosis (> G1), indwelling ureteral stent, and chemotherapy for recurrent disease. To address whether what type of urinary diversion increases the risk of postoperative stage 3B CKD, incontinent urinary diversion was divided into 2 groups, cutaneous ureterostomy and ideal conduit in the Cox regression model 2.

## Results

**Fig 1:**
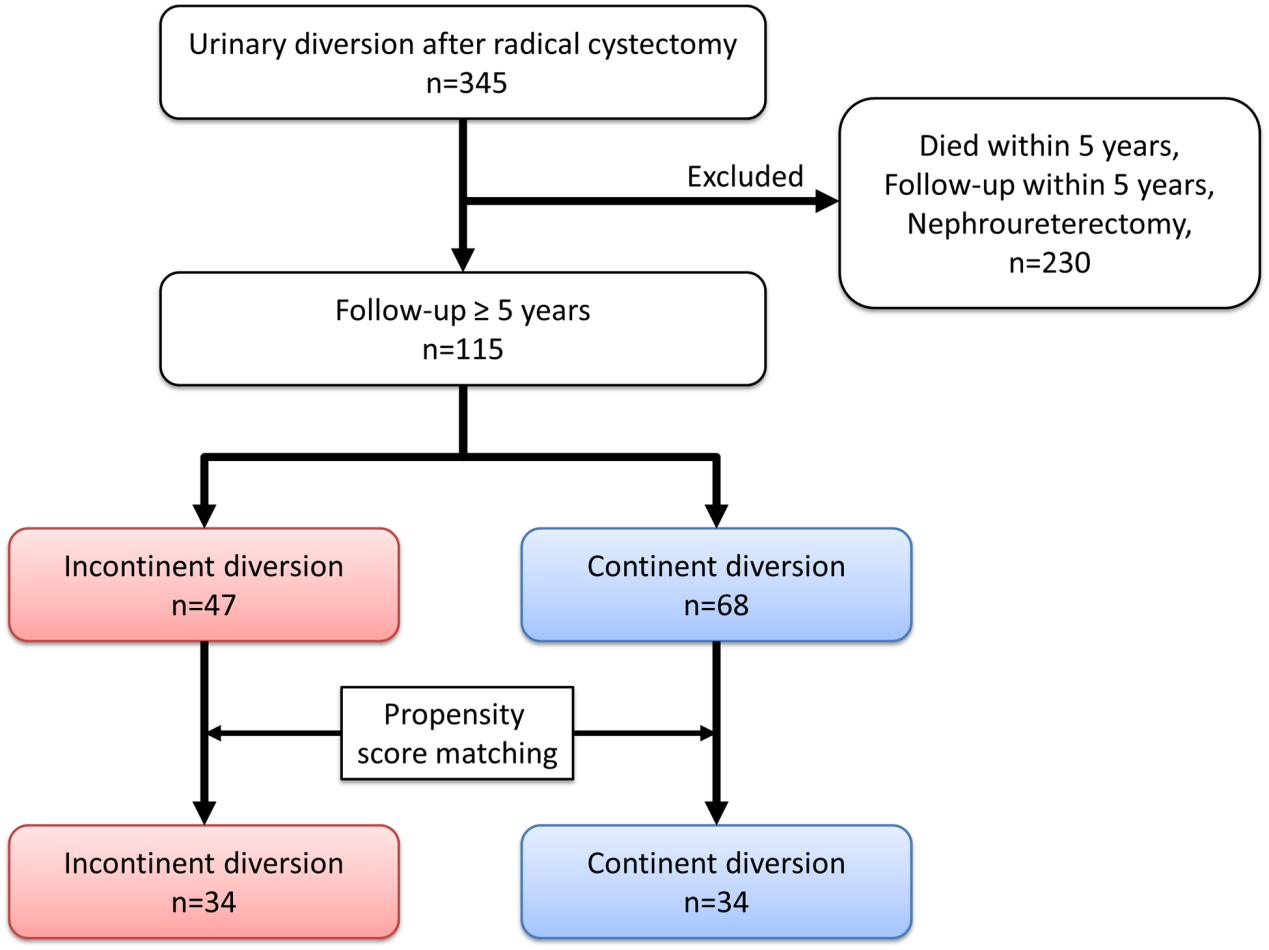
Patient selection and study design. We performed radical cystectomy and urinary diversion in 345 consecutive adult patients in our hospital. Of these, 115 patients with more than a 5-year follow-up were identified. Forty-seven patients were included the incontinent diversion group (ileal conduit or cutaneous ureterostomy), and 68 were included in the continent group (orthotopic ileal neobladder). Sixty-eight of these patients (*n* = 34 each) were selected using the propensity score matching method.

Patients’ clinicopathological characteristics and distributions are presented in Table 1 and Fig 1. A total of 47 patients underwent incontinent diversion, and 68 underwent continent diversion. In the incontinent group, ileal conduit diversion and cutaneous ureterostomy were performed in 17 and 30 patients, respectively. Demographic differences between the incontinent and continent groups were age at surgery, pathological T and N stage, postoperative complications, ureteral stent placement, and preoperative eGFR. Fifty-three patients (46%) received neoadjuvant chemotherapy. [26] All complications in this cohort were grade 1 or 2 in the Clavien classification.

**Table 1:** Clinical and pathological patient characteristics.

	All patients			Matched patients		
	Incontinent	Continent	*P value*	Incontinent	Continent	*P value*
n	47	68		34	34	
Ileal conduit / cutaneous ureterostomy, n =	17 / 30			14 / 20		
Median age*, years (IQR)	67 (64–76)	68 (58–71)	*0*.*018*	66 (61–72)	68 (58–71)	*0*.*437*
Gender* (M/F), n =	39 / 8	47 / 21	*0*.*126*	26 / 8	27 / 7	*0*.*770*
Mean ECOG-PS* (±SD)	0.0±0.2	0.0±0.0	*0*.*160*	0.1±0.2	0.0±0.0	*0*.*156*
Median follow-up, months (IQR)	72 (53–112)	97 (70–125)	*0*.*093*	73 (54–98)	101 (74–126)	*0*.*139*
Neoadjuvant chemotherapy, n =	18 (38%)	35 (51%)	*0*.*186*	13 (38%)	16 (47%)	*0*.*624*
TNM classification						
cT* (±SD)	2.4±0.9	2.3±0.8	*0*.*259*	2.4±0.9	2.2±0.8	*0*.*212*
pT (±SD)	2.1±1.2	1.4±1.2	*0*.*001*	2.0±1.2	1.3±1.1	*0*.*009*
pN+, n =	7 (15%)	1 (1.5%)	*0*.*008*	6 (18%)	1 (3%)	*0*.*105*
Cardiovascular disease*, n =	3 (6%)	9 (13%)	*0*.*354*	2 (6%)	1 (3%)	*0*.*555*
Hypertension*, n =	21 (45%)	27 (40%)	*0*.*701*	14 (41%)	14 (41%)	*1*.*000*
Diabetes*, n =	5 (11%)	8 (12%)	*1*.*000*	5 (15%)	6 (18%)	*0*.*742*
Median blood loss, kg (IQR)	1.5 (0.9–2.3)	1.3 (0.9–2.0)	*0*.*131*	1.4 (1.1–1.9)	1.3 (0.9–1.6)	*0*.*153*
Median operative duration, hours (IQR)	4.7 (4.0–6.3)	5.0 (4.4–6.2)	*0*.*897*	4.8 (4.0–6.2)	4.8 (4.5–6.0)	*0*.*789*
Postoperative Complications, n =	18 (38%)	26 (38%)	*1*.*000*	19 (56%)	19 (56%)	*1*.*000*
Postoperative hydronephrosis (> G1), n =	16 (34%)	5 (7%)	*<0*.*001*	12 (35%)	4 (12%)	*0*.*043*
Ureteral stent, n =	23 (49%)	0 (0%)	*<0*.*001*	15 (44%)	0 (0%)	*<0*.*001*
Ileal conduit, stent (+)	2 (12%)			1 (7%)		
Cutaneous ureterostomy, stent (+)	21 (70%)			14 (70%)		
Recurrence, n =	15 (32%)	6 (9%)	*0*.*003*	10 (29%)	2 (6%)	*0*.*023*
Chemotherapy for recurrent disease, n =	14 (30%)	4 (6%)	*0*.*001*	9 (27%)	1 (2.9%)	*<0*.*001*
Median cycle (IQR)	4 (2–6)	4 (1–5)	*0*.*413*	6 (3–14)	3 (1–4)	*0*.*029*

* applied for propensity score-matching

SD, standard deviation; IQR, interquartile range.

**Fig 2:**
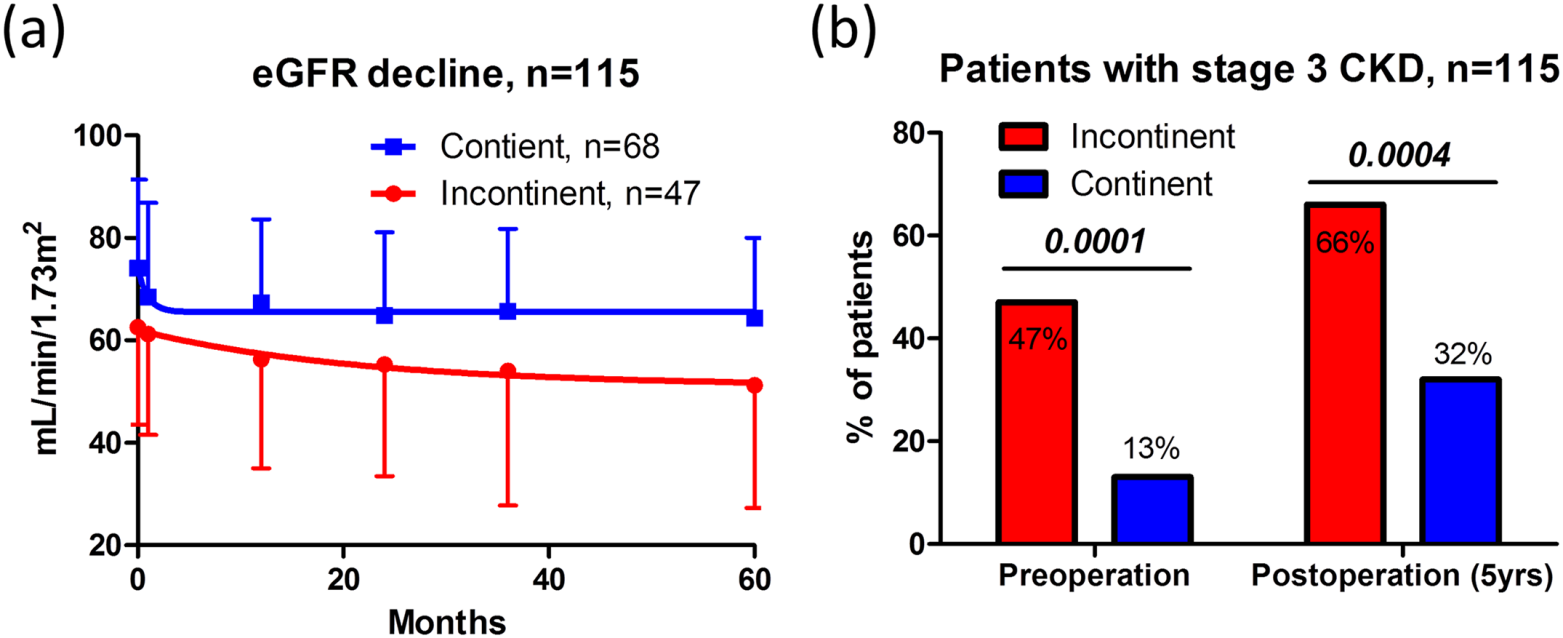
Pre- and postoperative renal function in all patients. The eGFR decline curve showed that pre- and postoperative eGFR were significantly lower in the incontinent group than in the continent group (unpaired *t*-test) (a). The proportion of patients with pre- and postoperative stage 3 CKD (eGFR < 60 mL/min/1.72 m^2^) was significantly different between the groups (Fisher’s exact test) (b).

The mean preoperative eGFR was significantly lower in the incontinent than in the continent group (*P* < 0.001, unpaired *t* test), and similar observations were recorded for postoperative eGFR at 5 years (Fig 2a). The number of patients with stage 3 CKD or before and after surgery was significantly higher in the incontinent than in the continent group (Table 2, Fig 2b).

**Table 2:** Renal function before and after surgery.

	All patients			Matched patients		
	Incontinent	Continent	*P value*	Incontinent	Continent	*P value*
eGFR (±SD)						
Before surgery*	63±19	74±17	*0*.*000*	69±18	70±17	*0*.*944*
After surgery						
1 month	61±19	69±18	*0*.*027*	67±18	67±18	*0*.*928*
1 year	56±22	67±16	*0*.*002*	62±21	65±16	*0*.*567*
5 years	51±24	64±16	*0*.*002*	57±25	60±17	*0*.*481*
eGFR decreasing rate (±SD)						
1 month (%)	1.5±21	6.7±20	*0*.*184*	2.0±20	2.0±19	*0*.*942*
5 years (%)	17±32	12±19	*0*.*334*	17±32	12±20	*0*.*448*
Stage ≥ 3 CKD, n =						
before surgery	22/47 (47%)	9/68 (13%)	*<0*.*001*	10/34 (29%)	8/34 (24%)	*0*.*582*
5 years after surgery	31/47 (66%)	22/68 (32%)	*<0*.*001*	18/34 (53%)	14/34 (41%)	*0*.*331*
Stage ≥ 3B CKD, n =						
before surgery	10/47 (21%)	2/68 (3%)	*0*.*003*	2/34 (6%)	2/34 (6%)	*1*.*000*
5 years after surgery	23/47 (49%)	13/68 (19%)	*<0*.*001*	15/34 (44%)	7/34 (21%)	*0*.*038*

* applied for propensity score-matching;

SD, standard deviation.

To control for differences in the backgrounds, we applied the propensity score matching strategy using logistic analysis. In propensity score-matched patients (*n* = 34 each), no significant differences were observed in patients’ background, except for pathological T stage, ureteral stent placement, and tumor recurrence. The number of patients with recurrence was 12, which was significantly larger in the incontinent group (*P* = 0.023). Of those, 10 patients received platinum based chemotherapy for recurrent disease. Majority of patients (70%) received carboplatin based chemotherapy due to the ineligibility for cisplatin. We experienced ureteroenteric stricture in 13 patients, urolithiasis in 2 patients, and postoperative hydronephrosis in 16 patients. No patient experienced stomal stenosis. The median renal function decline rates were 17.6%, 14.3%, and 32.5% in ureteroenteric stricture, urolithiasis and postoperative hydronephrosis, respectively. There were no significant differences in decline rates between the two groups.

**Fig 3:**
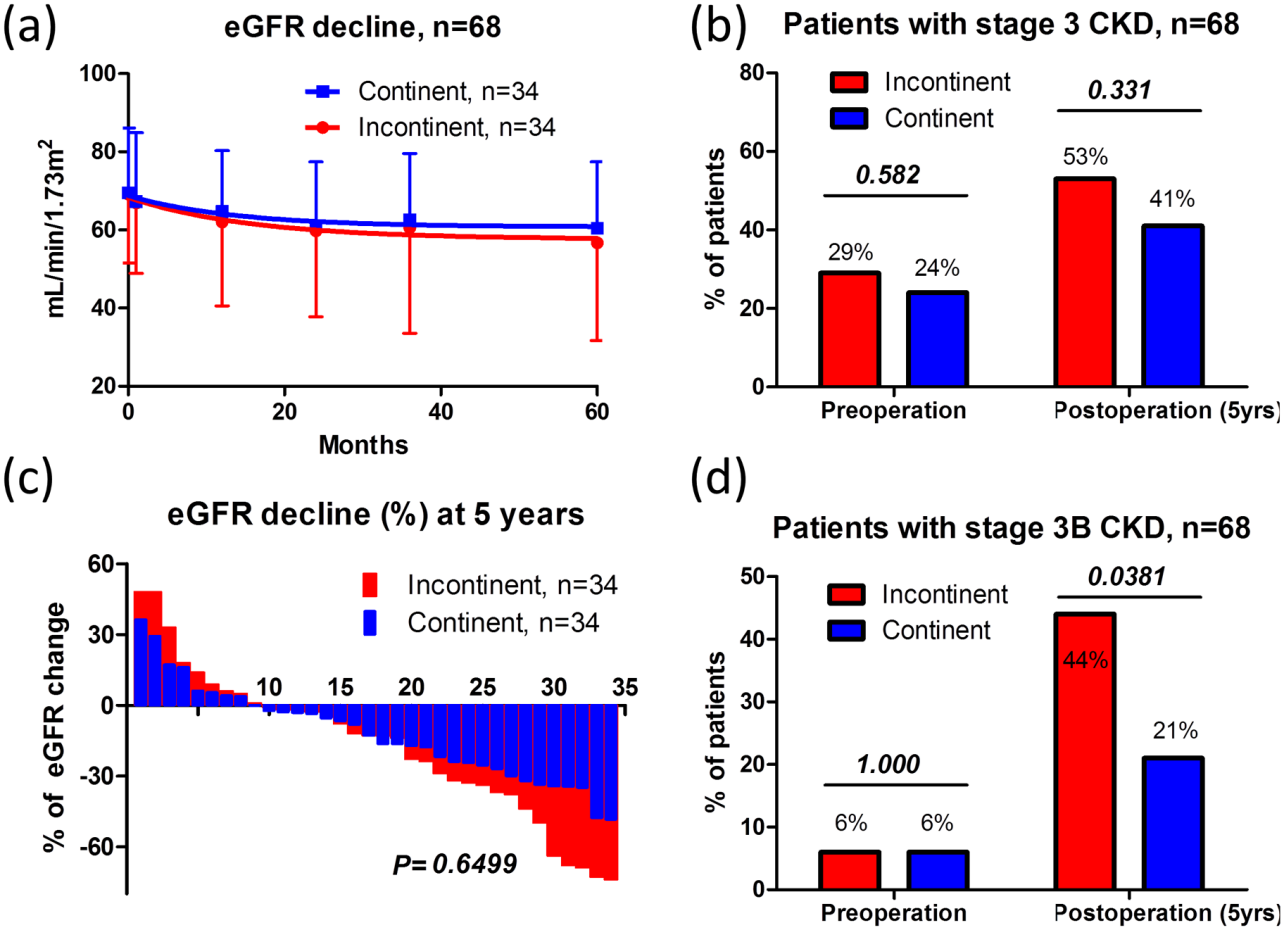
Pre- and postoperative renal function in propensity score-matched patients. No significant differences were observed in pre- and postoperative renal function after the 5-year follow-up (a). The proportion of patients with pre- and postoperative stage 3 CKD (eGFR < 60 mL/min/1.72 m^2^) was not significantly different between the groups (Fisher’s exact test) (b). A waterfall plot showed no significant differences in 5-year eGFR decrease rates after urinary diversion between the groups (unpaired *t*-test, *P* = 0.6499) (c). The proportion of patients with preoperative stage 3B CKD (eGFR < 45 mL/min/1.72 m^2^) was not significantly different between the groups; however, a significant difference was observed in the proportion of patients with postoperative stage 3B CKD in the incontinent group (Fisher’s exact test) (d).

In the pair-matched patients, no significant differences were observed between the groups in postoperative eGFR (Fig 3a), 5-year eGFR decrease rates, or the number of patients with stage 3 CKD at 5 years after surgery (Fig 3b). The mean 5-year eGFR decrease rate was 17 ± 32% and 12 ± 20% in the incontinent and continent groups, respectively (Table 2, *P* = 0.448, unpaired *t* test). We analyzed renal function decline between 1) ileal conduit and cutaneous ureterostomy, 2) ileal conduit and neobladder, 3) cutaneous ureterostomy and neobladder, separately. Median decline of eGFR between ileal conduit, cutaneous ureterostomy, and neobladder were 10.4%, 19%, and 14.6%, respectively. There were no differences in renal function decline between the groups; ileal conduit and cutaneous ureterostomy: 10.4% vs. 19% (*P* = 0.740), ileal conduit and neobladder: 10.4% vs 14.6% (*P* = 0.950), cutaneous ureterostomy and neobladder: 19% vs. 14.6%, (*P* = 0.513).

**Fig 4:**
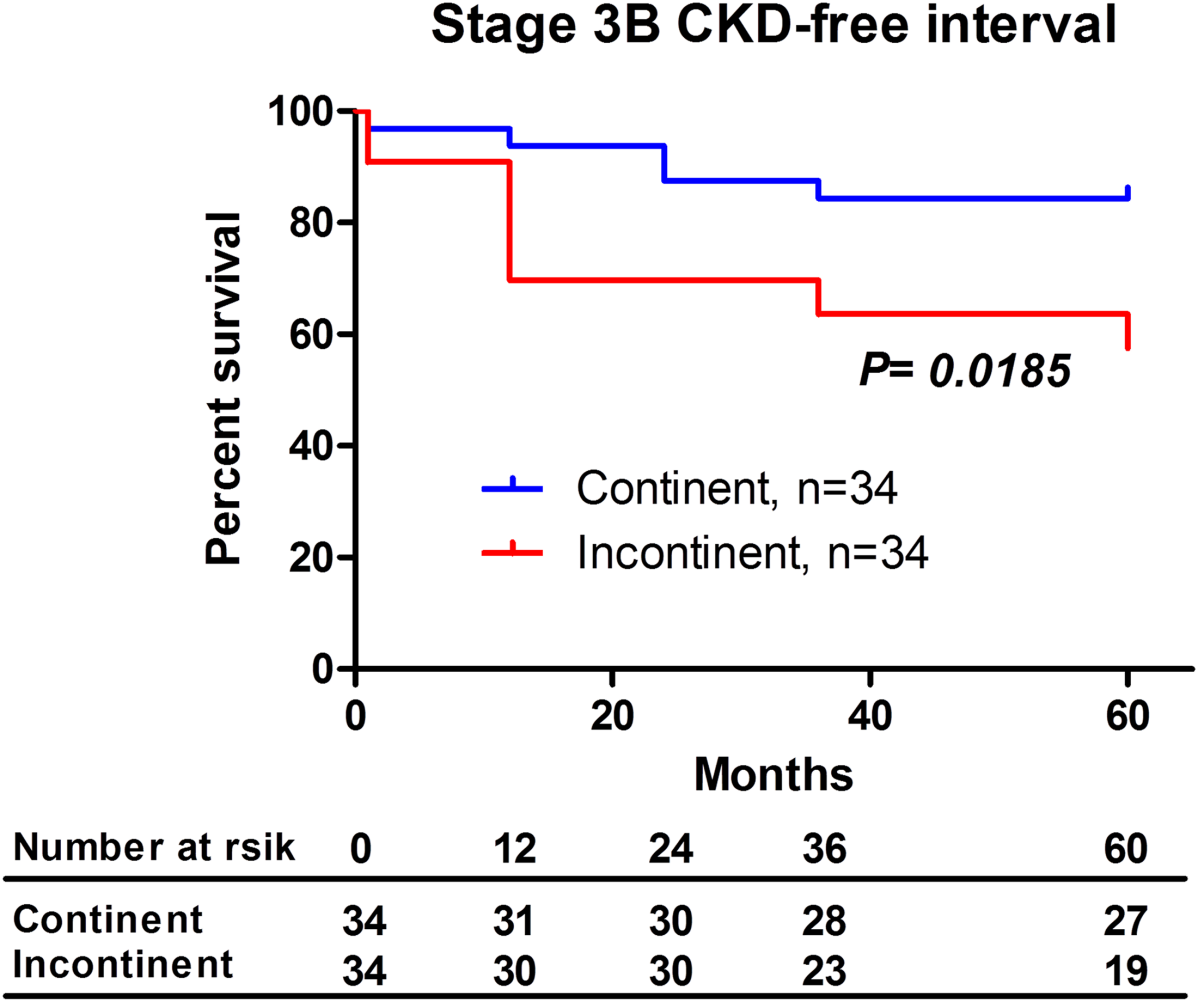
Stage 3B CKD-free interval rates in propensity score-matched patients. The Kaplan–Meier curve showed that 5-year stage 3B CKD-free interval rates were significantly higher in the continent group (84%) than in the incontinent group (58%) (Log-rank test).

**Fig 5:**
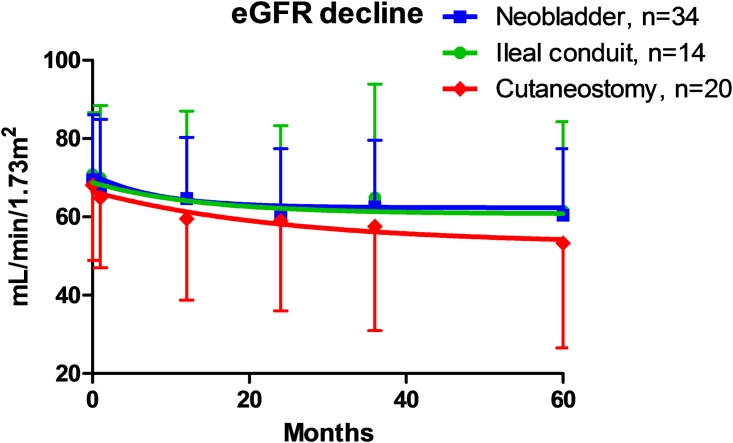
Pre- and postoperative renal function in propensity score-matched patients among 3 types of urinary diversion. Cutaneous ureterostomy showed tendency of renal function decline compared with other urinary diversion, although there were no significant differences in pre- and postoperative renal function after the 5-year follow-up (*P* = 0.473, One-way ANOVA analysis).

A waterfall plot for 5-year eGFR decrease rates after urinary diversion demonstrated no significant differences between the groups (Fig 3c, *P* = 0.650, unpaired *t* test). However, the number of patients with stage 3B CKD at 5 years after surgery was significantly higher in the incontinent group (Fig 3d, *P* = 0.038, chi-square test). The Kaplan–Meier curve showed that 5-year stage 3B CKD-free interval rates were significantly higher in the continent (84%) than in the incontinent group (58%) (Fig 4, *P* = 0.018, log–rank test). Using multivariate Cox regression analysis, potential risk factors significantly associated with stage 3B CKD after radical cystectomy and urinary diversion were elderly patient (> 67 years old), eGFR before surgery (< 60 ml/min/1.73m^2^), incontinent diversion, and postoperative hydronephrosis (> G1) (Table 3, model 1). To address the influence of urinary diversion on postoperative stage 3B CKD, incontinent diversion was separately analyzed by multivariate Cox regression analysis (model 2). In this model, cutaneous ureterostomy was selected as an independent risk factors for postoperative stage 3B CKD (Table 3, model 2). Cutaneous ureterostomy showed tendency of renal function decline compared with other urinary diversion, although there were no significant differences in pre- and postoperative renal function after the 5-year follow-up (*P* = 0.473, One-way ANOVA analysis). (Fig 5)

**Table 3:** Multivariate Cox regression analyses of risk factors for postoperative stage 3B CKD (eGFR < 45 mL/min/1.73 m^2^). Incontinent urinary diversion was divided into 2 groups, and compared with orthotopic ileal neobladder in the model 2. Comorbidities included past history of CVD, HTN, or DM.

Variables (Model 1)	Fisk factors	*P value*	HR	95%CI
Age	Older than 67 years	*0*.*043*	2.90	1.03–8.13
Comorbidities	Pre-existing	*0*.*110*	2.40	0.82–7.02
eGFR before surgery	< 60 ml/min/1.73m2	*0*.*020*	3.25	1.20–8.80
Stent indwelling	Positive	*0*.*163*	0.40	0.11–1.45
Postoperative hydronephrosis	Greater than grade 1	*0*.*002*	5.15	1.81–14.6
Tumor recurrence	Positive	*0*.*900*	0.91	0.22–3.71
Chemotherapy for recurrent disease	Positive	*0*.*290*	0.43	0.09–2.04
Urinary diversion	Incontinent diversion	*0*.*016*	4.41	1.33–14.7
Variables (Model 2)				
Age	Older than 67 years	*0*.*025*	3.47	1.17–10.4
Comorbidities	Pre-existing	*0*.*129*	2.32	0.78–6.87
eGFR before surgery	< 60 ml/min/1.73m2	*0*.*016*	3.32	1.25–8.82
Stent indwelling	Positive	*0*.*065*	0.22	0.04–1.10
Postoperative hydronephrosis	Greater than grade 1	*0*.*003*	4.81	1.70–13.7
Tumor recurrence	Positive	*0*.*969*	0.97	0.23–4.11
Chemotherapy for recurrent disease	Positive	*0*.*196*	0.33	0.06–1.77
Urinary diversion (ref. neobladder)	Cutaneous ureterostomy	*0*.*012*	8.92	1.63–48.7
	Ileal conduit	*0*.*069*	3.44	0.91–13.1

## Discussion

Urinary diversion after radical cystectomy is mandatory for muscle-invasive bladder cancer patients, and it should ensure protection of the upper urinary tract. Because a high proportion of urinary bladder cancer patients present with some degree of baseline renal impairment,[5, 27] follow-up of postoperative renal function is critical for long-term morbidity and mortality. However, a comparison of postoperative renal function is challenging because of selection bias for urinary diversion and the definition of renal function decline is controversial. Hautmann et al.[28] and Osaka et al.[12] defined a 25% decline in eGFR from baseline as renal function decline, and Jin et al. [10] and Eisenberg et al.[11] defined the decline as a 10% decline in eGFR from baseline. In contrast, Samuel et al.[13] defined an eGFR < 50 mL/min/1.73 m^2^ as renal function decline. Recently, Gershman et al. [14] reported approximately 70% of patients undergoing radical cystectomy with urinary diversion experienced renal function decline regardless of type of diversion. It is evident that a certain level of decline in renal function is expected after urinary diversion, but no clear evident threshold value has been determined for renal function decline after urinary diversion. Moreover, these studies were mainly reported from Western countries. Because Japanese people tend to be low in renal function compared with Caucasian people, [21] their findings need to be investigated in Japan.

The association between postoperative renal function and risks for progressive renal impairment or cardiovascular disease after radical cystectomy should be noted. Thus, we used the presence of stage 3B CKD as the threshold for renal function decline. Stage 3 CKD is used to indicate the status of moderately impaired kidney function in the present study. Stage 3 CKD was reclassified into 3A (eGFR = 45–59 mL/min/1.73 m^2^) and 3B (eGFR = 30–44 mL/min/1.73 m^2^). Individuals with stage 3B CKD are considered to have a much higher risk for progressive renal disease and cardiovascular disease compared with those with stage 3A CKD.[22] Based on this classification, we defined the presence of postoperative stage 3B CKD as a risk factor for renal function decline in Japanese patients.

Several risk factors have been reported for postoperative renal function decline. Recent retrospective studies suggested that urinary tract obstruction or stricture, postoperative pyelonephritis, diabetes, proteinuria and hypertension represent potentially modifiable factors associated with renal function decline.[10–14] The decision of urinary diversion had no effect on patients with a 10%–25% decline in postoperative renal function; however many confounding factors prevented a definite conclusion from being reached. To overcome this problem, we used statistical matching methods to reduce the preoperative patients’ background as much as possible. Similar to previous reports, we also evaluated risk factors for a 10% or 25% decline in eGFR at 5 years; however, no independent risk factors were identified for renal function decline (data not shown). Because the mean 5-year eGFR decrease rate was approximately 15% in this cohort, a 10% or 25% decline in eGFR may be not significant in the present study.

Our results showed that the types of urinary diversion had no significant effect on renal function decline; however, older age, preoperative impaired renal function, postoperative hydronephrosis, and incontinent diversion (cutaneous ureterostomy) were potential risk factors for postoperative stage 3B CKD. As previous studies suggested, the other potential risk factors for stage 3B CKD were ureteroenteric stricture, urolithiasis, chemotherapy for recurrent disease, respectively. In the present study, chemotherapy for recurrence had no impact on postoperative stage 3B CKD. Because majority of patients (70%) in this cohort received carboplatin based chemotherapy due to the ineligibility for cisplatin, it might not have significant impact on renal function. Based on these observations, our results suggests that the types of urinary diversion may not adversely affect a mild decline in renal function, while older age, preoperative impaired renal function, postoperative hydronephrosis, and cutaneous ureterostomy may increase the tendency to develop stage 3B CKD.

There is a question why cutaneous ureterostomy may increase the risk to develop stage 3B CKD. First, a potential reason is hydronephrosis that subsequent to ureteral stricture in incontinent diversion. Our data showed postoperative hydronephrosis was significantly frequent in incontinent diversion (Table 1). Because these factors are known as important prognostic factors for postoperative renal function outcome, we hypothesized indwelling ureteral stent due to postoperative ureteral obstructions has potential risk factor for renal function decline. However, it was not selected in multivariate analysis. Because our indication for indwelling ureteral stent is acute pyelonephritis and/or ureteral stenosis, obstructive events might improve by ureteral stenting. Based on this situation, non-urgent hydronephrosis that not required indwelling ureteral stent may play a key role for renal function decline. Second, in our technique of ileal neobladder replacement, a U-shaped neobladder is constructed using only 40 cm of detubularized ileum, which has lower storage pressure, leads to gradual improvement in the voiding profile with adequate reservoir capacity. [19, 20, 29, 30] These conditions might have protective effect on upper urinary tract, and prevent renal function deterioration. Third, because incontinent diversion tends to be selected in older patients, the risk for lower preoperative eGFR, advanced disease, or postoperative stage 3B CKD may be higher in the incontinent group. Therefore, even with the use of statistical matching technique, we could not exclude selection bias for urinary diversion.

The present study has several limitations including the small sample size, statistical power, its retrospective nature, and a sample composition that excluded many patients who were deceased within 5 years. We could not control selection bias and other unmeasurable confounding factors even using matching methods. In addition, we could not include well-known other clinical factors such as obesity, smoking habit, nutrition condition, and level of proteinuria due to unavailability of data. Despite these limitations, an advantage of this study was the use of propensity score matching between patients with incontinent and continent diversion. The data revealed no differences in postoperative renal function decline after radical cystectomy and urinary diversion, and renal function decline was noted in the majority of patients at the 5-year follow-up after radical cystectomy.

In conclusion, the present data suggest that the types of urinary diversion had no significant effect on renal function decline. However, elderly patients, preoperative impaired renal function, postoperative hydronephrosis, and incontinent diversion (cutaneous ureterostomy) were potential risk factors for stage 3B CKD. Therefore, caution for postoperative renal function should be exercised in patients who underwent a cutaneous ureterostomy. Further investigation by well-designed randomized prospective studies is necessary to assess the changes of postoperative renal function decline in patients with muscle-invasive bladder cancer.
