# Correction: Renal Function Outcomes and Risk Factors for Risk Factors for Stage 3B Chronic Kidney Disease after Urinary Diversion in Patients with Muscle Invasive Bladder Cancer

**DOI:** 10.1371/journal.pone.0151742

**Published:** 2016-03-15

**Authors:** Shingo Hatakeyama, Takuya Koie, Takuma Narita, Shogo Hosogoe, Hayato Yamamoto, Yuki Tobisawa, Tohru Yoneyama, Takahiro Yoneyama, Yasuhiro Hashimoto, Chikara Ohyama

The words “Risk Factors for” appear twice in the title of this article. The correct title is: Renal Function Outcomes and Risk Factors for Stage 3B Chronic Kidney Disease after Urinary Diversion in Patients with Muscle Invasive Bladder Cancer. The correct citation is: Hatakeyama S, Koie T, Narita T, Hosogoe S, Yamamoto H, Tobisawa Y, et al. (2016) Renal Function Outcomes and Risk Factors for Stage 3B Chronic Kidney Disease after Urinary Diversion in Patients with Muscle Invasive Bladder Cancer. PLoS ONE 11(2): e0149544. doi:10.1371/journal.pone.0149544

There are formatting errors in Table 1 and Table 3 of the published article. Please see the correct Table 1 and Table 3 here.

**Table 1:** Clinical and pathological patient characteristics.

	All patients			Matched patients		
	Incontinent	Continent	*P value*	Incontinent	Continent	*P value*
n	47	68		34	34	
Ileal conduit / cutaneous ureterostomy, n =	17 / 30			14 / 20		
Median age*, years (IQR)	67 (64–76)	68 (58–71)	*0*.*018*	66 (61–72)	68 (58–71)	*0*.*437*
Gender* (M/F), n =	39 / 8	47 / 21	*0*.*126*	26 / 8	27 / 7	*0*.*770*
Mean ECOG-PS* (±SD)	0.0±0.2	0.0±0.0	*0*.*160*	0.1±0.2	0.0±0.0	*0*.*156*
Median follow-up, months (IQR)	72 (53–112)	97 (70–125)	*0*.*093*	73 (54–98)	101 (74–126)	*0*.*139*
Neoadjuvant chemotherapy, n =	18 (38%)	35 (51%)	*0*.*186*	13 (38%)	16 (47%)	*0*.*624*
TNM classification						
cT* (±SD)	2.4±0.9	2.3±0.8	*0*.*259*	2.4±0.9	2.2±0.8	*0*.*212*
pT (±SD)	2.1±1.2	1.4±1.2	*0*.*001*	2.0±1.2	1.3±1.1	*0*.*009*
pN+, n =	7 (15%)	1 (1.5%)	*0*.*008*	6 (18%)	1 (3%)	*0*.*105*
Cardiovascular disease*, n =	3 (6%)	9 (13%)	*0*.*354*	2 (6%)	1 (3%)	*0*.*555*
Hypertension*, n =	21 (45%)	27 (40%)	*0*.*701*	14 (41%)	14 (41%)	*1*.*000*
Diabetes*, n =	5 (11%)	8 (12%)	*1*.*000*	5 (15%)	6 (18%)	*0*.*742*
Median blood loss, kg (IQR)	1.5 (0.9–2.3)	1.3 (0.9–2.0)	*0*.*131*	1.4 (1.1–1.9)	1.3 (0.9–1.6)	*0*.*153*
Median operative duration, hours (IQR)	4.7 (4.0–6.3)	5.0 (4.4–6.2)	*0*.*897*	4.8 (4.0–6.2)	4.8 (4.5–6.0)	*0*.*789*
Postoperative Complications, n =	18 (38%)	26 (38%)	*1*.*000*	19 (56%)	19 (56%)	*1*.*000*
Postoperative hydronephrosis (> G1), n =	16 (34%)	5 (7%)	*<0*.*001*	12 (35%)	4 (12%)	*0*.*043*
Ureteral stent, n =	23 (49%)	0 (0%)	*<0*.*001*	15 (44%)	0 (0%)	*<0*.*001*
Ileal conduit, stent (+)	2 (12%)			1 (7%)		
Cutaneous ureterostomy, stent (+)	21 (70%)			14 (70%)		
Recurrence, n =	15 (32%)	6 (9%)	*0*.*003*	10 (29%)	2 (6%)	*0*.*023*
Chemotherapy for recurrent disease, n =	14 (30%)	4 (6%)	*0*.*001*	9 (27%)	1 (2.9%)	*<0*.*001*
Median cycle (IQR)	4 (2–6)	4 (1–5)	*0*.*413*	6 (3–14)	3 (1–4)	*0*.*029*

* applied for propensity score-matching SD, standard deviation; IQC, interquartile range.

**Table 3:** Multivariate Cox regression analyses of risk factors for postoperative stage 3B CKD (eGFR < 45 mL/min/1.73 m^2^). Incontinent urinary diversion was divided into 2 groups, and compared with orthotopic ileal neobladder in the model 2. Comorbidities included past history of CVD, HTN, or DM.

Variables (Model 1)	Fisk factors	*P value*	HR	95%CI
Age	Older than 67 years	*0*.*043*	2.90	1.03–8.13
Comorbidities	Pre-existing	*0*.*110*	2.40	0.82–7.02
eGFR before surgery	< 60 ml/min/1.73m2	*0*.*020*	3.25	1.20–8.80
Stent indwelling	Positive	*0*.*163*	0.40	0.11–1.45
Postoperative hydronephrosis	Greater than grade 1	*0*.*002*	5.15	1.81–14.6
Tumor recurrence	Positive	*0*.*900*	0.91	0.22–3.71
Chemotherapy for recurrent disease	Positive	*0*.*290*	0.43	0.09–2.04
Urinary diversion	Incontinent diversion	*0*.*016*	4.41	1.33–14.7
**Variables (Model 2)**				
Age	Older than 67 years	*0*.*025*	3.47	1.17–10.4
Comorbidities	Pre-existing	*0*.*129*	2.32	0.78–6.87
eGFR before surgery	< 60 ml/min/1.73m2	*0*.*016*	3.32	1.25–8.82
Stent indwelling	Positive	*0*.*065*	0.22	0.04–1.10
Postoperative hydronephrosis	Greater than grade 1	*0*.*003*	4.81	1.70–13.7
Tumor recurrence	Positive	*0*.*969*	0.97	0.23–4.11
Chemotherapy for recurrent disease	Positive	*0*.*196*	0.33	0.06–1.77
Urinary diversion (ref. neobladder)	Cutaneous ureterostomy	*0*.*012*	8.92	1.63–48.7
	Ileal conduit	*0*.*069*	3.44	0.91–13.1
